# Single-cell RNA sequencing in atherosclerosis: Mechanism and precision medicine

**DOI:** 10.3389/fphar.2022.977490

**Published:** 2022-10-04

**Authors:** Qiaoyu Li, Mengchen Wang, Shuxia Zhang, Meiqi Jin, Rongchang Chen, Yun Luo, Xiaobo Sun

**Affiliations:** ^1^ Institute of Medicinal Plant Development, Peking Union Medical College and Chinese Academy of Medical Science, Beijing, China; ^2^ Beijing Key Laboratory of Innovative Drug Discovery of Traditional Chinese Medicine (Natural Medicine) and Translational Medicine, Beijing, China; ^3^ Key Laboratory of Bioactive Substances and Resource Utilization of Chinese Herbal Medicine, Ministry of Education, Beijing, China; ^4^ NMPA Key Laboratory for Research and Evaluation of Pharmacovigilance, Beijing, China

**Keywords:** atherosclerosis, single-cell RNA sequencing, heterogeneity, precision medicine, therapeutic target

## Abstract

Atherosclerosis is the pathological basis of various vascular diseases, including those with high mortality, such as myocardial infarction and stroke. However, its pathogenesis is complex and has not been fully elucidated yet. Over the past few years, single-cell RNA sequencing (scRNA-seq) has been developed and widely used in many biological fields to reveal biological mechanisms at the cellular level and solve the problems of cellular heterogeneity that cannot be solved using bulk RNA sequencing. In this review, we briefly summarize the existing scRNA-seq technologies and focus on their application in atherosclerosis research to provide insights into the occurrence, development and treatment of atherosclerosis.

## 1 Introduction

Atherosclerosis is the pathological basis of a variety of vascular diseases, which can involve multiple organs, such as the heart and brain, leading to coronary heart disease, stroke, and peripheral artery disease. Ischemic heart disease and stroke are the leading causes of death worldwide (Roth et al., 2020). Additionally, as an atherosclerotic disease, peripheral artery disease has high prevalence, and is associated with high cardiovascular mortality (Campia et al., 2019; Bevan and White Solaru, 2020). Therefore, it is important to elucidate the pathogenesis of atherosclerosis. Several studies have elucidated some of these underlying mechanisms. Endothelial dysfunction due to factors, such as hemodynamic forces and reduced nitric oxide bioavailability, is the initial stage of atherosclerosis (Landmesser et al., 2000; Favero et al., 2014; Gordon et al., 2020). Low density lipoprotein (LDL) accumulates in intima due to endothelial dysfunction, and is modified to oxidized LDL (oxLDL) (Wolf and Ley, 2019). This process activates endothelial cells (ECs) which express cell adhesion molecules (like vascular cell adhesion molecules-1, intercellular adhesion molecule-1, P-selectin and E-selectin) and inflammatory factors (like interleukin-8), recruiting monocytes and lymphocytes to the intima and promoting inflammation (Hermida and Balligand, 2014; Kattoor et al., 2017; Mundi et al., 2018; Zhu et al., 2018). Monocytes differentiate into macrophages in the intima, which plays an important role in the progression of atherosclerosis (Tabas and Bornfeldt, 2016). Macrophages take up oxLDL and become foam cells with cholesterol accumulation (Yu et al., 2013). In addition, a small number of foam cells are derived from the transformation of vascular smooth muscle cells (VSMCs) in response to the stimulation of disease environment (Marchio et al., 2019). Cholesterol loading causes secretion of pro-inflammatory cytokine, proliferation, and further recruitment of myeloid cells, which further promotes inflammation (Kattoor et al., 2017; Wolf and Ley, 2019). Other types of immune cells, such as neutrophils, T cells and B cells, are also involved in the inflammatory process. As the disease progresses, macrophage apoptosis and impaired efferocytosis promote the development of plaque which is covered by a fibrous cap of VSMCs that migrate to the lumen side (Linton et al., 2016). Plaque expansion leads to narrowing of vascular lumen, which causes ischemia of surrounding tissues and organs. Vulnerable plaque rupture and atherothrombosis may cause acute ischemic syndromes (Badimon and Vilahur, 2014). However, the occurrence and development of atherosclerosis involve many types of cells, which makes the mechanism very complex, and its pathogenesis is still not fully understood.

Recently, single-cell RNA sequencing (scRNA-seq) technology has been developed. Traditional bulk RNA sequencing results reflect the average level of RNA in the sequenced samples, while scRNA-seq can detect the RNA information of a single cell, identify expression differences between different types of cells, and reveal cell heterogeneity within a tissue to provide more precise and detailed transcription information (Lafzi et al., 2018). At present, scRNA-seq technology is widely used in many biological fields, such as developmental biology, immunology, neurobiology, and cancer research (Kolodziejczyk et al., 2015).

ScRNA-seq provides new insights into the pathogenesis and treatment of atherosclerosis. This review briefly summarizes the existing scRNA-seq technologies and focuses on the application of scRNA-seq technology in atherosclerosis research in recent years (Supplementary Table S1).

## 2 ScRNA-seq methods and platforms

**FIGURE 1:**
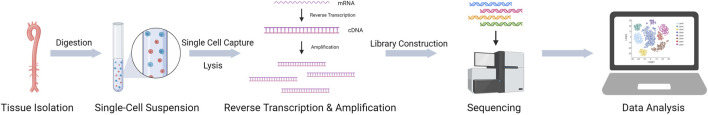
Basic steps of scRNA-seq.

The first scRNA-seq study was published in 2009 (Tang et al., 2009). With the development and optimization of technology and the reduction of sequencing costs, a variety of scRNA-seq methods have been developed, including Smart-seq, Smart-seq2, CEL-seq, and Drop-seq (Hedlund and Deng, 2018). Some commercial platforms have been developed based on these methods. The main commercial scRNA-seq platforms are the 10X Genomics Chromium™ System, BD Rhapsody™ Single-Cell Analysis System, and Fluidigm C1 System. Each of these methods has its own characteristics, and previous studies have compared multiple methods, which facilitates the selection of an appropriate method (Ziegenhain et al., 2017). In general, scRNA-seq includes four steps (Figure 1): 1) single-cell or single-nucleus sample preparation, 2) reverse transcription, 3) cDNA library amplification, and 4) sequencing library preparation and sequencing (Hedlund and Deng, 2018). In addition, subsequent data analysis is important. Using scRNA-seq data enables a variety of unique analytical options, such as constructing a cell atlas, studying cell-cell interactions, and revealing cell developmental trajectories through pseudotime analysis (Chaudhry et al., 2019; Zheng et al., 2020). Here, we will briefly introduce two common scRNA-seq methods.

### Drop-seq

Drop-seq is a droplet-based high-throughput scRNA-seq method (Macosko et al., 2015). In this method, single cells and microparticles with special primers are separated into microdroplets using a microfluidic device to form an independent reaction chamber. Primer sequences include the PCR handle, cell barcode, unique molecular identifier, and poly (dT) sequences. Cells are lysed in the droplets, and mRNA is captured by poly (dT) on microparticles. The microparticles are then collected, washed, and subjected to reverse transcription and PCR amplification, and a cDNA library is established for sequencing. Drop-seq can achieve high-throughput cell sequencing, but the sequencing depth is low, making it suitable for transcriptome sequencing analysis of a large number of cells with low sequencing depth. Similar to Drop-seq, 10X Genomics Chromium is a commercial drop-based scRNA-seq platform (Zhang et al., 2019) that is the most widely used in atherosclerosis research, as summarized in (Supplementary Table S1).

### Smart-seq

Smart-seq is a robust scRNA-seq method that significantly increases transcriptome coverage (Ramsköld et al., 2012). The primer containing oligo (dT) binds to the polyA sequence of RNA and is reverse-transcribed under the action of Moloney murine leukemia virus reverse transcriptase to form the first strand, and three cytosine residues (C) are attached at the 5 ′end of the first strand. The primer containing poly-G binds to the three cytosine residues of the first strand to synthesize the second strand. PCR-amplified cDNA is used for sequencing. The Fluidigm C1 system uses the Smart-seq method and automates Smart-seq steps (See et al., 2018). Smart-seq2 is optimized and improved from Smart-seq (Picelli et al., 2014). Both Smart-seq and Smart-seq2 can generate full-length cDNA, have high transcriptome coverage, and detect single nucleotide polymorphisms and mutations, but the cell throughput is low (Ramsköld et al., 2012; Picelli et al., 2014).

Although scRNA-seq has provided new and more precise insights into many biological fields, the technology currently has some limitations. The cost of scRAN-seq has decreased with technological development and the emergence of commercial platforms; however, it is still relatively expensive, compared with bulk RNA sequencing, which limits its application. The preparation of scRNA-seq samples has high requirements to ensure the survival and good state of the cells. To obtain cell suspension, tissue generally is cut into small segments and digested with enzyme cocktails at 37°C, which may lead to artifactual changes in gene expression as cells are surrounded by a strange environment and the enzymes within the cells are also maximally active at this temperature. This problem can be ameliorated and resolved by the adoption of cold-adapted proteases that have high activity at low temperatures, which allows digestion to be performed on ice or at low temperatures (Adam et al., 2017). In addition, during the process of cell suspension preparation and single cell capture, dead cells as well as the presence of doublets (droplets with two or more cells) can lead to artifacts that affect subsequent data analysis. Generally, dead cells can be removed to a large extent by sorting or dead cell removal kit, though there will be some cells lost at the same time. The presence of doublets can be solved to some extent by sample multiplexing approaches (Kang et al., 2018; Stoeckius et al., 2018; McGinnis et al., 2019b; Gaublomme et al., 2019; Shin et al., 2019), and McGinnis et al. (McGinnis et al., 2019a) developed a computational doublet detection tool, Doublet-Finder, which can be applied to existing scRNA-seq datasets, overcoming the limits of sample multiplexing. Furthermore, although scRNA-seq can cluster cells based on differences in transcriptional information, it still relies on known cellular markers for cell identification. Therefore, cells with several types of markers could cause certain difficulties in confirming cell identity. These problems can be solved better by advances in technology and analytical methods.

## 3 ScRNA-seq applications in atherosclerosis research

The pathogenesis of atherosclerosis is complex and involves various cells. With the development of technology in recent years, scRNA-seq has been used to construct an atherosclerosis cell atlas of mice and humans, including immune cells, such as monocytes/macrophages, dendritic cells, T cells, B cells, natural killer (NK) cells, and non-immune cells, such as VSMCs, ECs, pericytes, and fibroblasts (Cochain et al., 2018; Kim et al., 2018; Winkels et al., 2018; Gu et al., 2019; Depuydt et al., 2020; Burger et al., 2022). Research has revealed cellular heterogeneity and provided new insights into the pathogenesis of atherosclerosis. The content and results of these studies have mainly focused on immune cells (monocytes/macrophages and T cells), VSMCs, and ECs, contributing to the determination of their role in atherosclerosis.

### 3.1 Endothelial cells

Endothelial dysfunction is a critical early step in atherosclerosis development (Hansson, 2005). Mechanical sensors in ECs can identify blood flow, thereby regulating gene expression, endothelial function, and atherogenic pathways. Atherosclerosis preferentially occurs in arterial areas affected by disturbed flow (d-flow), whereas arterial areas affected by stable flow (s-flow) are protected (Chiu and Chien, 2011; Kwak et al., 2014; Tarbell et al., 2014).

To further study the mechanism, by which d-flow induces atherosclerosis through regulating gene expression in ECs, Andueza et al. (Andueza et al., 2020) used a mouse partial carotid artery ligation (PCL) model, and scRNA-seq was performed on ECs enriched from the left and right carotid arteries exposed to d-flow and s-flow. This study showed that d-flow induced EC transformation into pro-inflammatory cells, endothelial-to-mesenchymal transition (EndMT) cells, hematopoietic stem cells, endothelial stem/progenitor cells, and an unexpected immune cell-like (EndICLT) phenotype. D-Flow reprograms ECs from atheroprotective to pro-atherogenic phenotypes. Moreover, (Williams et al., 2022) analyzed the data from Andueza et al. (Andueza et al., 2020) and found that Klk10 expression in ECs was regulated by blood flow, with high expression in s-flow and low expression in d-flow, and that Klk10 inhibited endothelial inflammation and atherosclerosis. In another d-flow-related study, (Li F. et al., 2021) also found that d-flow induced EC transformation into pro-atherogenic phenotypes. Several EC subsets have been identified, which highly express Klk8 (a secretory serine protease, acting as an oncogene or anti-oncogene in various tumors and associated with learning and memory), Lrp1(LDL receptor-related protein 1, associated with lipid homeostasis, clearance of apoptotic cells and metabolism of amyloid-β peptides), Dkk2 (dickkopf WNT signaling pathway inhibitor 2, related to angiogenesis) and Cd36 (a scavenger receptor associated with lipid metabolism) respectively, namely Klk8^hi^, Lrp1^hi^, Dkk2^hi^, and Cd36^hi^ ECs (Endemann et al., 1993; Min et al., 2011; Shinohara et al., 2017; Li J. et al., 2021; Hua et al., 2021). Klk8^hi^ and Lrp1^hi^ ECs are mainly derived from non-PCL carotid arteries, whereas Dkk2^hi^ and Cd36^hi^ ECs are d-flow-derived EC subsets. Dkk2^hi^ ECs are mechanosensitive and highly express genes related to atherosclerosis (*Ngf, Col8a1, Kit,* and *Vcan*), and they may be transformed from Klk8^hi^ ECs under d-flow conditions. Significantly enriched genes in Cd36^hi^ECs (*Cd36*, *Gpihbp1*, *Scarb1*, and *Abca1*) were associated with lipid metabolism and storage.

In a study on diabetes-related atherosclerosis, (Zhao et al., 2021) identified eight EC subpopulations, three of which expressed mesenchymal markers, indicating EndMT and showing a fibroblast-like phenotype. EndMT-derived fibroblast-like cells exhibit genetic characteristics associated with ECM organization, apoptosis, and cytokine production, which are important in atherosclerosis.

**FIGURE 2:**
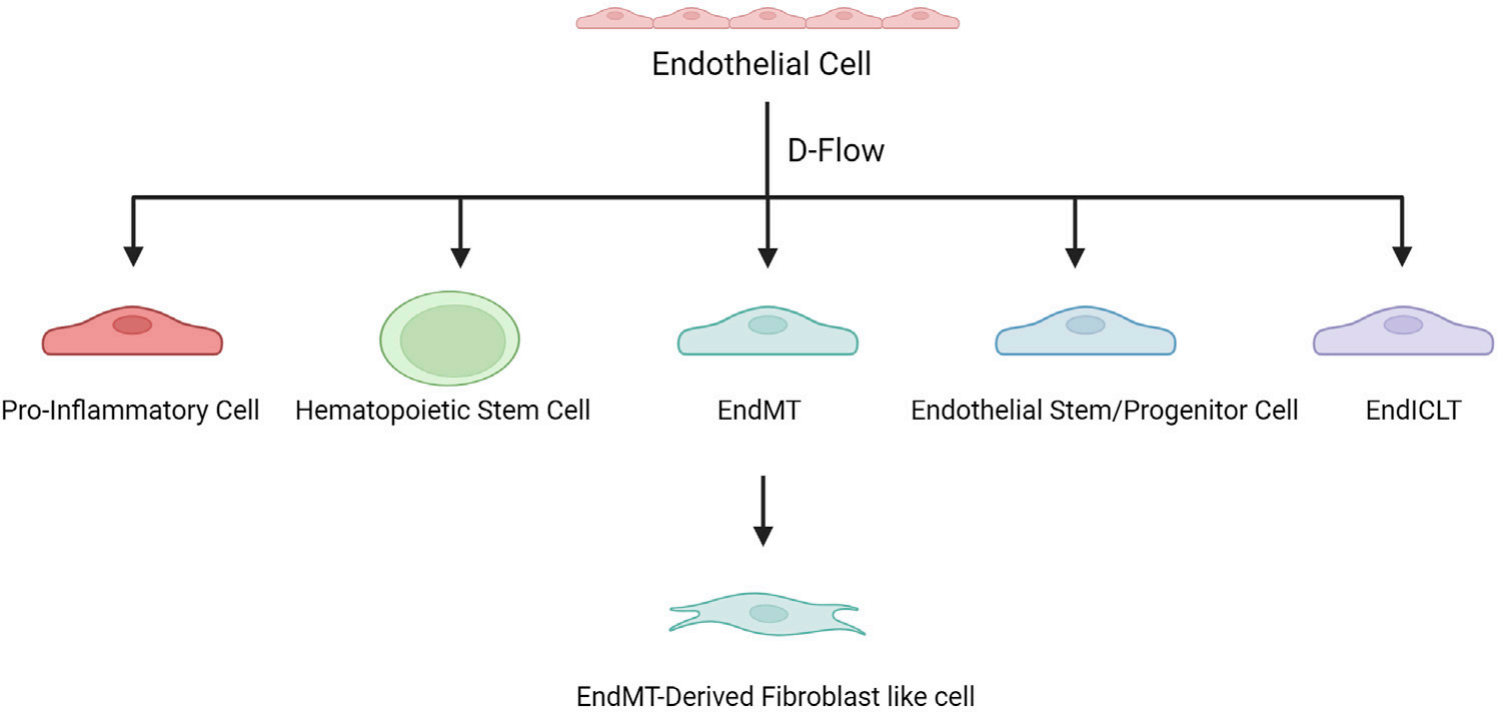
D-flow induces EC recoding to produce multiple proatherogenic phenotypes.

These studies have revealed the plasticity and heterogeneity of ECs in atherosclerosis. D-flow is associated with endothelial dysfunction at the onset of atherosclerosis and induces EC recoding to produce multiple proatherogenic phenotypes that contribute to disease progression (Figure 2).

### 3.2 Monocytes/macrophages

Atherosclerosis is a chronic inflammatory disease, where monocytes and macrophages play important roles. Macrophages are the primary immune cells in atherosclerotic lesions (Tabas and Bornfeldt, 2016). Most macrophages in the plaque originate from monocytes in the blood recruited during disease progression (Moore et al., 2013).

There are two main monocyte subpopulations in mouse blood: Ccr2^+^Cx3cr1^+^ (Ly6C^hi^) classical monocytes and Ccr2^-^Cx3cr1^++^ (Ly6C^lo^) patrolling nonclassical monocytes, which have obvious migratory and inflammatory properties (Geissmann et al., 2010). Ly6C^hi^ monocytes are thought to become classically activated macrophages (M1 macrophages) under most inflammatory conditions, often referred to as inflammatory monocytes (Nahrendorf et al., 2007; Tacke et al., 2007; Auffray et al., 2009). However, a study by (Rahman et al., 2017) has shown that Ly6C^hi^ monocytes can also be recruited and polarized into M2-like macrophages during atherosclerotic plaque regression. This indicated that Ly6C^hi^ monocytes can generate macrophages with different phenotypes under different physiological and environmental stimuli.

Barrett et al. (2019) found that platelets induce monocyte migration and recruitment into atherosclerotic plaques, leading to plaque platelet-macrophage aggregation. ScRNA-seq of the aortic arch of hypercholesterolemic mice showed increased expression of the platelet-specific transcripts platelet factor 4 (Pf4) and pro-platelet basic protein (Ppbp) in the plaque macrophage population, suggesting increased macrophage-platelet aggregation in plaques. In addition, the expression of suppressor of cytokine signaling 3 (SOCS3) increased in macrophages, and the SOCS1:SOCS3 ratio decreased. Platelet-induced SOCS3 expression regulates the plaque macrophage phenotype and promotes atherosclerosis by promoting the production of inflammatory cytokines (IL6, IL1b, and TNFa) and impairing phagocytosis.

Lin et al. (2019) applied scRNA-seq to plaque cells derived from CX3CR1^+^ precursors of the aortic arch during atherosclerosis progression and regression in mice. The results showed that the macrophage activation state spectrum was more complex than that of the M1 and M2 polarization states, whereas atherosclerosis progression was associated with differentiation into a more distinct macrophage state than that during resolution. In addition, a cluster of proliferating monocytes with stem-like features was detected, suggesting that these monocytes may still be in a proliferating, self-renewing state, rather than differentiating into macrophages immediately after entering the tissue.

Cochain et al. (2018) performed scRNA-seq on CD45^+^ cells from the aortas of non-diseased and atherosclerotic LDL receptor–deficient (Ldlr^−/−^) mice. Among the 13 cell subsets, there were five myeloid cell subsets, including monocytes, monocyte-derived dendritic cells, and three macrophage subsets. Resident-like macrophages were found in both healthy and atherosclerotic aortas, while the other two macrophage subsets were almost exclusively detected in atherosclerotic aortas, namely inflammatory macrophages with high IL1b expression and previously undescribed TREM2^hi^ macrophages that highly expressed triggering receptor expressed on myeloid cells 2 (TREM2). TREM2 is a member of the immunoglobulin superfamily and forms complex with TYRO protein tyrosine kinase-binding protein at cell surface which regulates the activation of macrophages, dendritic cells, osteoclasts and microglial cells (Roussos et al., 2015; Zhang et al., 2018). TREM2^hi^ macrophages appeared to be related to lipid metabolism and catabolism, suggesting that they were foam macrophages. These three macrophages are derived from CX3CR1^+^ monocyte precursors (Lin et al., 2019). Similarly, (Depuydt et al., 2020) found two pro-inflammatory macrophages expressing IL1b or TNF in human carotid plaques, as well as a foam cell-like group expressing TREM2, which showed a profibrotic phenotype. In addition, in a study on d-flow, (Li F. et al., 2021) identified 10 cell groups related to d-flow, including resident-like, TREM2^hi^, and Birc5^hi^ macrophages. Birc5^hi^ macrophages show high proliferative capacity, which is considered a potential factor in macrophage aggregation in atherosclerosis, and they may transform into dendritic cells.

Gu et al. (2019) performed scRNA-seq on the aortic adventitia of wild-type (WT) and apolipoprotein E-deficient (ApoE^−/−^) mice. ScRNA-seq results revealed a cellular atlas of the aortic adventitia, revealing a stem/progenitor-like and pro-inflammatory mesenchymal cluster II (Mesen II). In ApoE^−/−^ mice, resident macrophages were activated and associated with increased myeloid cell infiltration in the adventitia. Furthermore, cellular communication analysis revealed enhanced interactions between ApoE^−/−^ outer membrane mesenchymal clusters and inflammatory macrophages.

Schlegel et al. (2021) conducted scRNA-seq on mouse aortic immune cells before and after Netrin-1 (Ntn1) silencing and detected one monocyte population and two macrophage populations (Cx3cr1^−^ macrophages and TREM2^hi^ macrophages). Ntn1 is described as an axon guidance molecule, which also plays an important role in atherosclerosis by regulating inflammation and the migration of immune cells such as macrophages (Serafini et al., 1996; van Gils et al., 2012). Ntn1 silencing reorganizes the immune cell landscape in the arterial wall, reduces inflammation, increases the expression of M2 marker genes, and upregulates the gene pathways related to phagocytosis and migration in monocytes and TREM2^hi^ macrophages, including the C-C chemokine receptor type 7 signaling pathway required for macrophage migration from plaques and atherosclerosis regression, which suggested that targeting Ntn1 may improve atherosclerotic inflammation and promote plaque regression.

**FIGURE 3:**
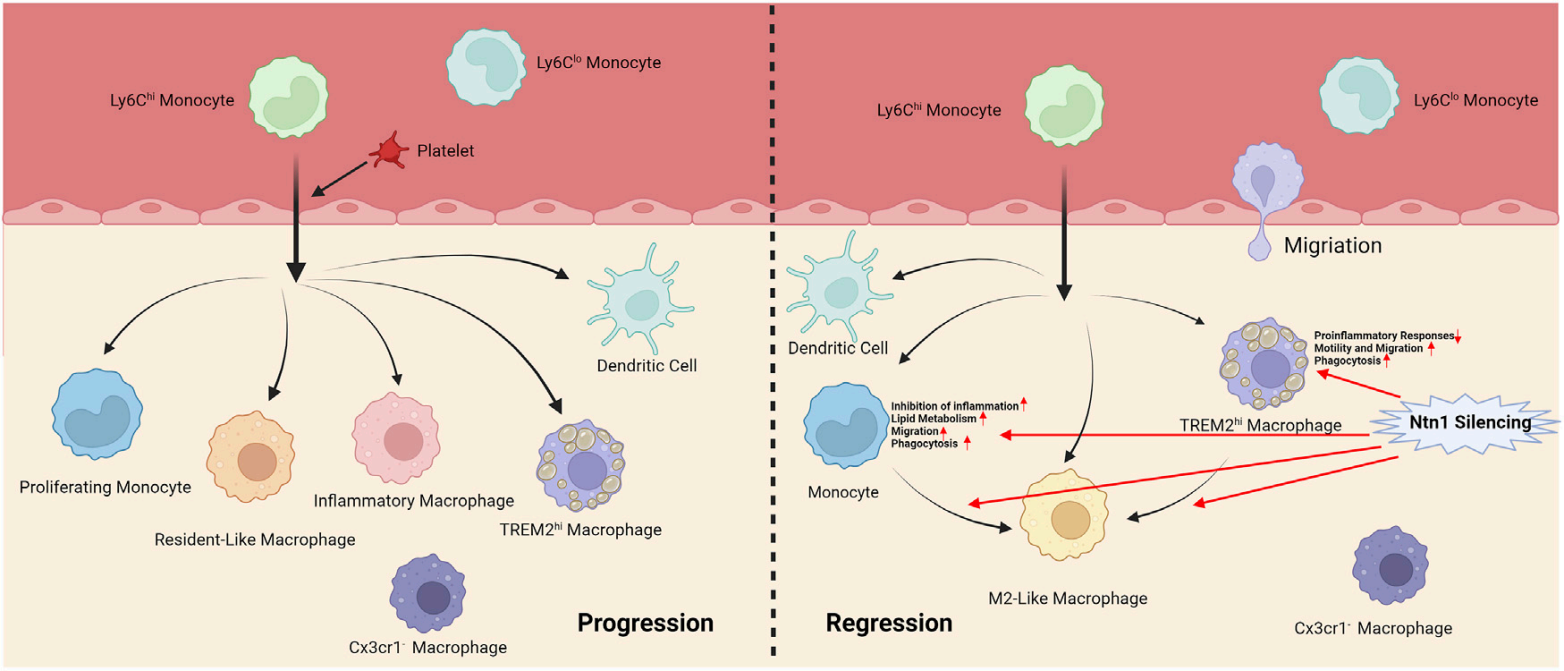
Different phenotypes of monocytes and macrophages during progression and regression of atherosclerosis.

The above studies have shown that platelets participate in the process of monocyte recruitment into arteriosclerotic plaque and regulate macrophage phenotypes; after monocytes enter the plaque, they differentiate into a variety of macrophages that play different roles under the stimulation of the physiological environment. Among them, the inflammatory macrophages TREM2^hi^ and Birc5^hi^ are associated with disease. Ntn1 silencing upregulates gene pathways related to macrophage phagocytosis and migration and promotes plaque regression. Furthermore, M2-like macrophages, which may be partially derived from Ly6C^hi^ monocytes, play an important role in atherosclerosis regression. These results provide new insights into the role of monocytes and macrophages in disease development and regression (Figure 3).

### 3.3 Lymphocyte

Recent studies have shown that T cells and B cells are key drivers and modulators of atherosclerosis (Wolf and Ley, 2019; Saigusa et al., 2020; Pattarabanjird et al., 2021). ScRNA-seq has contributed to evaluating their role in atherosclerosis.

#### 3.3.1 T cells

Winkels et al. (2018) identified several clusters of T cells, including CD4^+^ T cells (memory T cells, T_H_2 cells and T_H_17 cells), CD8^+^ T cells and mixed CD4^+^/CD8^+^ T cells through scRNA-seq conducted on aortas of ApoE^−/−^ mice with chow diet (CD) or western diet (WD). T_H_17 cells have been mostly considered as a pro-atherogenic phenotype. CD4^+^/CD8^+^ T cells, memory T cells, and CD8^+^ T cells transformed to a more pro-inflammatory phenotype in WD-fed mice. Depuydt et al. (2020) detected several clusters of CD4^+^ and CD8^+^ T cells with different activation states from a cytotoxic to a more quiescent phenotype in human carotid atherosclerotic plaques.

Sharma et al. (2020) found that increased regulatory T cells (Tregs) are a common feature of plaque regression in multiple mouse models of atherosclerosis. ScRNA-seq applied to plaque immune cells found that Tregs in progressive plaques highly expressed the thymus-derived or natural Treg marker Nrp1. Conversely, Tregs in regressing plaques showed lower Nrp1 mRNA levels and higher mRNA levels of factors related to the induction of Treg differentiation or maintenance (Mif, LGALs9, and Ly6a), suggesting that Tregs in regressive plaques may originate from the peripheral differentiation of naive T cells. Pathway analysis showed that Treg phenotype in regression changed, showing increased metabolic activity, increased lymphocyte reaction, and inhibition of apoptosis, cell movement, and glycosphingolipid metabolism compared with the progression phenotype. In addition, Tregs affected the macrophage inflammatory gene pathway and macrophage activation during regression.

Wolf et al. (2020) found atherosclerosis-protective ApoB-specific (ApoB^+^) CD4^+^ T cells with a Treg phenotype in healthy individuals. ScRNA-seq revealed that expanded plaque T cells consistently exhibited a mixed T_H_1/T_H_17 phenotype during atherosclerosis, whereas ApoB^+^ T cells formed several clusters with mixed T_H_ signalling. In atherosclerosis, ApoB^+^ T cells increased and gradually transformed into pathogenic T_H_1/T_H_17-like cells with pro-inflammatory properties, with only a residual Treg transcriptome.

#### 3.3.2 B cells

Winkels et al. (2018) filtered cellular *Cd19*-mRNA^+^ events from atherosclerotic aortas induced by WD and identified three subsets of B cells. The expression of genes associated with antigen-presentation, co-stimulation, antibody generation, and cellular-adhesion and cross-talk increased in B-cell cluster 1. Cluster 2 expressed cell-division genes, and apoptosis-related and pro-inflammatory TNF-signaling genes were up-regulated in Cluster 3. In addition, these three subsets could be distinguished by CD43 and B220 (CD43^high^B220^neg^, CD43^neg^B220^high^, CD43^low^B220^high^), and they showed differential expression of GM-CSF and CCL5, two important mediators of atherosclerosis. It was confirmed by quantitation of *in vitro* cytokine production that CD43^high^ B220^neg^ B cells expressed higher level of CCL5, while the other two clusters of B cells secreted the proatherogenic mediators IFN-γ and GM-CSF. There are other studies that detected B cells through scRNA-seq (Cochain et al., 2018; Gu et al., 2019; Depuydt et al., 2020), but they did not delve further into the phenotype and function of B cell populations.

### 3.4 Smooth muscle cells

VSMCs may play a role in fibrous caps and potentially necrotic cores during atherosclerosis through a process called “phenotypic transition” (Gomez and Owens, 2012; Bennett et al., 2016).

Dobnikar et al. (2018) performed scRNA-seq and lineage tracing of healthy mouse blood vessels to analyze VSMC heterogeneity. A rare group of VSMCs was detected, which expressed the multipotent progenitor cell marker Sca1/Ly6a, gradually downregulated the contraction gene, and upregulated the genes responding to inflammation and growth factors, suggesting that Sca1^+^ VSMC lineage cells in healthy tissues may represent a more plastic state, which can easily respond to injury and inflammation. Further studies showed that Sca1 upregulation was a marker of VSMCs experiencing phenotypic transformation *in vitro* and *in vivo* and revealed the same population of Sca1-positive VSMC lineage cells in atherosclerotic plaques. Pan et al. (2020) also found that SMCs can be transformed into intermediate cell states in mouse and human atherosclerotic plaques, namely “SEM” cells (expressing the stem cell, endothelial, and monocyte/macrophage markers, respectively Ly6a, Vcam1, and Ly6c1), which can differentiate into macrophage-like cells and fibrochondrocyte-like cells, or return to the SMC phenotype. In addition, the study showed that retinoic acid (RA) signaling was identified as a regulator of SMC-to-SEM cell transition. These two studies illustrate the existence of an intermediate SMC state in atherosclerosis, which contributes to the phenotypic transition.

Zhang Z. et al. (2022) analyzed the scRNA-seq data of human plaques from the carotid artery (GSE159677). The expression of lysosome- and inflammation-related genes increased in VSMCs derived from macrophage-like cells, and *NOTCH* genes negatively correlated with macrophage genes, suggesting their important role in VSMC transition to macrophage-like cells. In addition, *in vitro* studies have shown that inhibition of *NOTCH* signaling leads to dedifferentiation of VSMCs, resulting in complete transformation into macrophage-like cells.

Wirka et al. (2019) analyzed the transcriptome of modulated SMCs in mouse and human atherosclerotic arteries using scRNA-seq. This study showed that *TCF21* (a causal coronary artery disease gene) was specifically upregulated in SMCs during phenotypic regulation. These modulated SMCs expressed fibroblast-related genes and transformed into unique fibroblast-like cells named “fibromyocytes”. SMC-specific knockout of *TCF21* significantly inhibited SMC phenotype regulation in mice. Furthermore, TCF21 expression was closely related to regulation of the SMC phenotype in human diseased coronary arteries, and increased TCF21 expression was associated with a reduced risk of coronary artery disease in human coronary artery disease-associated tissues. Newman et al. (2021) reported that most protective ACTA2^+^ myofibroblast (MF)-like cells in fibrous caps originate from SMCs, and SMC-MF transition is induced by platelet-derived growth factor (PDGF) and transforming growth factor-β(TGF-β) and depends on aerobic glycolysis. ScRNA-seq of mouse brachiocephalic artery (BCA)lesions performed by Hartmann et al. (2021) showed that loss of hyaluronan synthase 3 (Has3) in SMCs enhanced SMC transformation to the ECM-producing Lgals3^+^ (galectin-3) phenotype, with increased acute phase response gene expression. Galectin-3(Gal3), a member of the galectin family of carbohydrate binding proteins, is involved in numerous biological activities such as cell growth, pre-mRNA splicing, inflammation and fibrosis, and is considered as a marker of damaged late endosomes (Papadopoulos and Meyer, 2017; Dong et al., 2018).

Alencar et al. (2020) revealed striking similarities in transcriptome clusters between mouse and human lesions, as well as extensive plasticity in SMCs and ECs. In ECs, KLF4 is a pulsatile shear stress (PS)-induced signal-dependent transcription factor, and is essential for endothelial lineage. Klf4 upregulates many atheroprotective genes, such as endothelial nitric oxide synthase (eNOS) and thrombomodulin under PS (Zhou et al., 2012; Fan et al., 2017; He et al., 2019). In addition, Klf4 is up-regulated in SMCs in response to vascular injury, and regulates many SMC genes, suggesting its role of phenotypic transition of SMCs (Yan et al., 2008). Klf4 regulates the transition to multiple phenotypes, including Lgals3^+^ osteoblasts, which may be detrimental to the pathogenesis of advanced atherosclerotic plaque. Lgals3 activation appears to be a marker of an early transitional state, where cells may subsequently exhibit multiple distinct phenotypes that develop at least three other SMC phenotypes in advanced lesions, such as the Klf4-dependent osteogenic phenotype associated with plaque calcification and plaque destabilization. Brandt et al. (2022) performed scRNA-seq on the aortic arch and root and descending thoracic aortas of ApoE^−/−^ mice and revealed five VSMC phenotypes, including the macrophagic calcific, mesenchymal chondrogenic, inflammatory and fibrous, inflammatory, and preserved contractile phenotypes. Growth differentiation factor 10 (GDF10) was co-expressed with markers of VSMCs transition to the osteo/chondrogenic phenotype (Runx2, osteopontin, and alkaline phosphatase), suggesting its promoting effect on the phenotypic transition. *In vitro* experiments confirmed that GDF10 promoted VSMC transformation into an osteogenic phenotype. Li F. et al. (2021) discovered a d-flow-dependent osteogenic phenotype, Spp1^hi^SMC, with functions, such as osteoblast differentiation, collagen biosynthesis, and vascular remodeling, which may play a role in d-flow-induced arterial stiffness. A study by (Burger et al., 2022) showed that Lyve-1 resident-like macrophages promoted VSMC transition into osteogenic-like cells by secreting CCL24. In addition, (Kim et al., 2020) performed scRNA-seq on mouse aortic roots and showed that SMC-specific deletion of the aryl hydrocarbon receptor (AHR) resulted in increased expression of chondrocyte markers (Col2a1 and Alpl) in regulated SMC chondrocytes.

Hartman et al. (2021) performed scRNA-seq on human atherosclerotic plaques and found that the key drivers of active gene regulatory networks in female coronary artery disease reside primarily in SMCs. Moreover, scRNA-seq data from WT and *Klf4*-knockout SMC lineage mice revealed that key female drivers of SMC were associated with Klf4, suggesting that sex differences in atherosclerosis are associated with phenotypic transformation of plaque SMCs (Alencar et al., 2020; Hartman et al., 2021).

**FIGURE 4:**
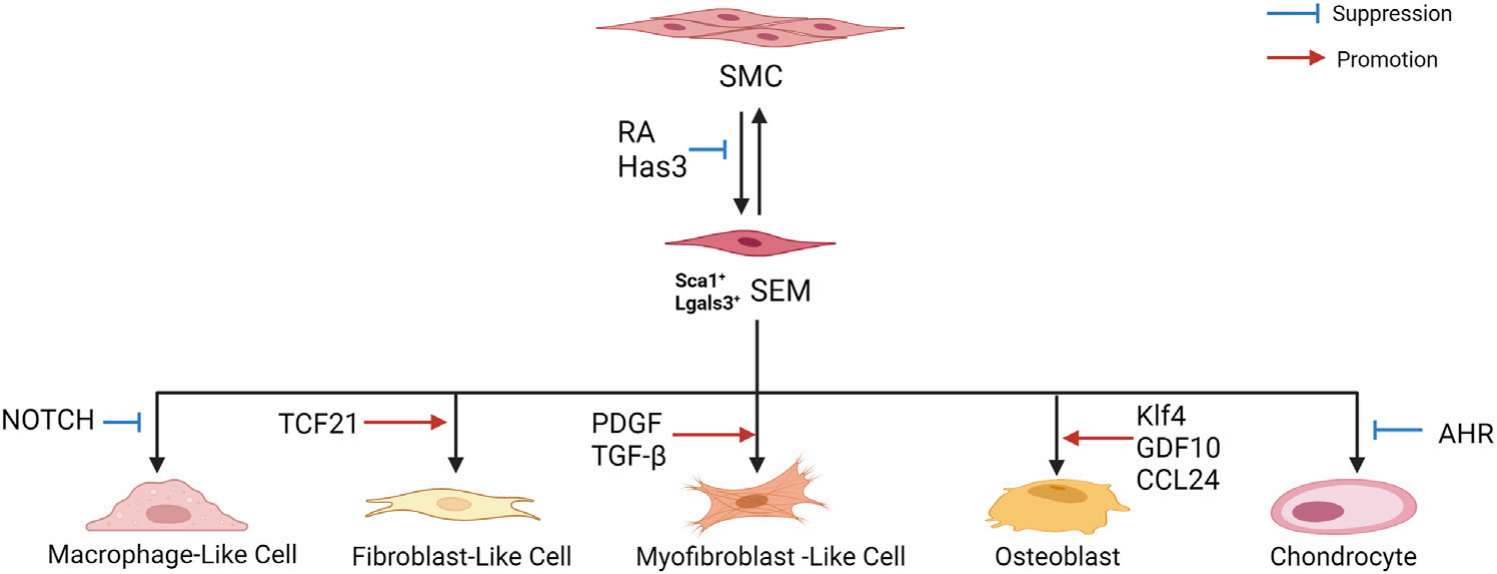
Phenotypic transition of SMCs in atherosclerosis.

The above studies indicate that in the process of atherosclerosis, SMCs assumed an intermediate state and a transition state expressing stem cell markers and then differentiate into macrophage-like, fibroblast-like, MF-like, and osteogenic/chondroid cells (Figure 4). In addition, they can return to the SMC phenotype. Fibroblast-like and MF-like cells are associated with fibrous cap stabilization, and osteoblast/chondroid-like cells are associated with calcification and vascular stiffness.

## 4 ScRNA-seq in atherosclerosis precision medicine

Research on the human genome and diseases has been based on the tissue level. The emergence of single-cell sequencing with higher resolution at the cellular level has undoubtedly promoted the progress of precision medicine. In atherosclerosis studies, scRNA-seq provides potential targets and contributes to drug discovery, thereby supporting clinical precision therapy.

### 4.1 Clinical diagnosis

In an atherosclerotic plaque visualization study based on positron emission tomography imaging, (Varasteh et al., 2021) explored the feasibility of targeting Gal3 expression to trace atherosclerosis using a zirconium-89 labeled Gal3-F (ab')2 monoclonal antibody. ScRNA-seq results showed that both M1 and M2 macrophages expressed Lgals3/Gal3 and that the Gal3 expression pattern in human plaques was similar to that in mouse plaques. This result supports the feasibility of the proposed method. In addition, clinical translation of this technology will assist in the diagnosis and imaging of atherosclerotic plaques at different stages, thereby developing personalized treatment plans.

### 4.2 Drug discovery

In recent years, advances have been made in nucleic acid-based therapies for atherosclerosis, including antisense oligonucleotides (Mäkinen et al., 2020). Zhang X. et al. (2022) delivered microRNA-33-5p (miR-33) antisense oligonucleotides to atherosclerotic plaques through pH-low-insertion peptide (pHLIP) constructs, avoiding the detrimental effects of total silencing of miR-33. ScRNA-seq showed that, compared with Scr^pHLIP^ (nontargeting antisense oligonucleotide conjugated with pHLIP) treatment, anti-miR-33^pHLIP^ increased the percentage of ECM^high^ Mac (expressing monocyte and macrophage genes and ECM-associated genes) and stem-like macrophages and decreased the percentage of inflammatory macrophages. Anti-miR-33^pHLIP^ upregulated fibrosis, M2 polarization, and antigen presentation pathways. The expression of fibrotic genes (*Col2a1, Col3a1, Col1a2,* and *Fn1*) and *Timp3* increased in macrophages from aortas from anti-miR-33^pHLIP^–treated mice, whereas Mmp12 expression decreased. These results indicated that anti-miR-33^pHLIP^ induces macrophages to a more stable phenotype in atherosclerosis and promotes its regression.

In a nanoparticle study, (Flores et al., 2020) developed macrophage-specific nanotherapy based on single-walled carbon nanotubes (SWNTs) containing an inhibitor of the anti-phagocytic CD47-SIRPα signaling axis. These SWNTs accumulated in atherosclerotic plaques, reactivated lesional phagocytosis, and reduced the plaque burden in ApoE^−/−^ mice. ScRNA-seq results demonstrated that prophagocytic SWNTs reduced the expression of inflammatory genes associated with the cytokine and chemokine pathways in diseased macrophages, suggesting their potential role in the prevention of atherosclerotic cardiovascular disease.

In these two studies, drugs targeted atherosclerotic lesions. ScRNA-seq revealed the effects of targeted drugs on specific cell populations and their precise mechanisms, providing theoretical support for their further clinical application.

### 4.3 Potential therapeutic targets

ScRNA-seq provides new insights into the mechanisms of atherosclerosis, as well as potential therapeutic targets. Ntn1 inhibition ameliorates atherosclerotic inflammation and promotes plaque regression (Schlegel et al., 2021). RA signaling, NOTCH, Tcf21, Has3, Klf4, GDF10, and AHR are associated with SMC phenotypic transition, and modulating these potential targets could affect disease-related cellular phenotypes, thereby exerting atheroprotective effects (Wirka et al., 2019; Alencar et al., 2020; Kim et al., 2020; Pan et al., 2020; Hartman et al., 2021; Hartmann et al., 2021; Newman et al., 2021; Brandt et al., 2022; Zhang Z. et al., 2022). The discovery of these potential targets provides new ideas and directions for the development of new atherosclerotic drugs.

## 5 Summary and future prospects

Atherosclerosis is a potential cause of high-mortality diseases, such as ischemic heart disease and stroke. Although there are many studies on atherosclerosis, its mechanism still requires further exploration owing to its complexity. Various cells are involved in the occurrence, development, and regression of atherosclerosis. Studying their roles and interactions is vital to revealing the pathogenesis and therapeutic targets of atherosclerosis. The emergence of scRNA-seq has provided great assistance in related research.

ScRNA-seq has revealed cellular heterogeneity in atherosclerosis. Disease-relevant cell types identified by analyzing cellular composition in health and disease, such as inflammatory macrophages and various d-flow-induced cells, including EndMT cells, may be potential targets for disease prevention and treatment (Cochain et al., 2018; Andueza et al., 2020). Using scRNA-seq, new cell subsets, such as TREM2^hi^ macrophages associated with lipid metabolism, catabolism, and fibrosis were discovered (Cochain et al., 2018). ScRNA-seq results also showed that M2-like macrophages and Tregs play a promoting role during the regression process (Rahman et al., 2017; Sharma et al., 2020). Furthermore, scRNA-seq combined with lineage tracing technology revealed the remarkable plasticity of VSMCs and ECs. Under stimulation by disease factors, VSMCs and ECs generate cells with different phenotypes, which play different roles in various diseases. VSMCs generate macrophage-, fibroblast-, and osteoblast-like cells through phenotypic transitions during the disease process (Wirka et al., 2019; Pan et al., 2020). ECs are transformed into pro-inflammatory cells, EndMT cells, hematopoietic stem cells, endothelial stem/progenitor cells, and EndICLT upon d-flow induction (Andueza et al., 2020). In addition, research has provided potential therapeutic targets and new ideas for the treatment of atherosclerosis, including Ntn1, NOTCH, and Tcf21.

In addition to the cells mentioned above, scRNA-seq also detected some other cells during atherosclerosis in the studies we summarized, such as dendritic cells, NK cells, granulocytes, but their functions and phenotypes were not delved in depth, which may provide directions for future atherosclerosis-related scRNA-seq studies (Cochain et al., 2018; Kim et al., 2018; Depuydt et al., 2020).

Currently, scRNA-seq has provided much support for the study of the mechanism of atherosclerosis, but it is less used to study drug mechanisms of action. ScRNA-seq has been used in only a few studies on targeted drugs for the treatment of atherosclerosis (Flores et al., 2020; Zhang X. et al., 2022). However, because scRNA-seq can analyze genetic information at the individual cell level to provide researchers with more detailed and comprehensive information, it also has advantages for drug research. ScRNA-seq can identify cell types associated with drug action and show the effects on different cells *in vivo*, providing more precise targets for drug research, which is not possible with previous sequencing technologies. Therefore, it has broad application prospects in drug research, especially for traditional Chinese medicines with complex mechanisms. For example, (Tian et al., 2022) recently revealed that the Qing-Fei-Pai-Du decoction can ameliorate disease-induced changes in cell composition, gene expression, and core transcriptional regulatory networks and correct abnormal cell-to-cell communication patterns.

With the further technological development and continuous reduction in cost, the application of scRNA-seq will become increasingly extensive. ScRNA-seq has revealed the heterogeneity of cell composition and function in atherosclerosis, provided more accurate insights into the mechanisms of diseases, and identified key targets. In addition, it can be combined with other single-cell -omics technologies to reveal the pathological mechanism of disease at different levels, clarify the mechanism of action of drugs, provide clinical diagnostic markers, assist in drug research, and develop personalized treatment plans to promote the progress of precision medicine.
