# Supplementary material for: Single-cell RNA sequencing in atherosclerosis: Mechanism and precision medicine
Source: Front Pharmacol. 2022 Oct 4;13:977490. doi: 10.3389/fphar.2022.977490 (PMC9576927; doi:10.3389/fphar.2022.977490)
Supplement: Supplementary file 1 [file Table1.docx]

Supplementary Material

**Table 1.** Application of scRNA-seq in atherosclerosis research.

| **Species** | **Tissue & Cell** | **Preparation for ScRNA-seq** | **Platform** | **Findings** | **Functional Phenotyping**  **(*In Vivo*, *Ex Vivo* or *In Vitro*)** | **Reference** |
| --- | --- | --- | --- | --- | --- | --- |
| Mouse | Cells of aortic adventitia from C57BL/6J mice and ApoE^-/-^ mice | Separation of adventitia.  Tissue was digested in Hank balanced salt solution (HBSS) containing 2 mg/mL Collagenase I and 2 mg/mL Dispase II for 30 minutes. | 10x Genomics GemCode | The landscape of adventitial cells: mesenchymal cells, immune cells (macrophages, T cells, and B cells), and some rare cells.  Activated resident macrophages in ApoE^-/-^ adventitia were associated with increased myeloid cell infiltration.  Enhanced interaction between a mesenchyme cluster and inflammatory macrophages in ApoE^-/-^ adventitia. | Transwell assay confirmed the proinflammatory role of SCA1^+^ Mesen II population.  Immunostaining confirmed the existence of rare cells. | (Gu et al., 2019) |
| Human | Cells from human carotid atherosclerotic plaques | Separation of plaques.  Tissue was digested in RPMI 1640 containing 2.5 mg/mL Collagenase Ⅳ, 0.25 mg/mL DNase Ⅰ, 2.5 mg/mL Human Albumin Fraction Ⅴ and 1 mM Flavopiridol at 37℃ for 30 minutes.  Cell suspension was filtered through a 70 µm strainer，and washed with RPMI 1640. | CEL-Seq2 | Fourteen distinct cell populations.  Subsets within EC populations showed angiogenic capacity and signs of EndMT.  CD4^+^ and CD8^+^ T cells showed activation-based subclasses.  Myeloid cells included two pro-inflammatory macrophage subsets and a foam cell-like population expressing TREM2 and showing a fibrosis-promoting phenotype. | Immunostaining confirmed that an EC subset express ACTA2 which is a typical SMC marker.  Flow cytometry confirmed the cytotoxic characteristics of CD4^+^CD28^null^ cells, and indicated that there were significantly more CD4^+^CD28^−^ cells than CD4^+^CD28^+^ cells | (Depuydt et al., 2020) |
| Mouse | CD45^+^ cells of whole aortas from ApoE^-/-^ mice and Ldlr^-/-^ mice | Aortas were separated and cut into small pieces.  Tissue was digested in HBSS containing 450 U/mL Collagenase Ⅰ, 250 U/mL Collagenase Ⅺ, 120 U/mL Hyaluronidase, and 120 U/mL DNase Ⅰ at 37℃ for 1 hour.  The suspension was filtered through a 50 µm strainer.  Fluorescence-activated cell sorting (FACS) sorted CD45^+^ cells. | 10x Genomics Chromium | Eleven main leukocyte clusters with distinct phenotypic and spatial characteristics.  Three major B-cell subsets altered in surface markers, functional pathways, and *in vitro* cytokine secretion. | Mass cytometry confirmed the phenotypical heterogeneity of aortic leukocytes.  It was confirmed by quantitation of *in vitro* cytokine production that CD43^high^ B220^neg^ B cells expressed higher level of CCL5, while the other two clusters of B cells secreted the proatherogenic mediators IFN-γ and GM-CSF. | (Winkels et al., 2018) |
| Mouse | CD45^+^ cells of whole aortas from ApoE^-/-^ mice and Ldlr^-/-^ mice | Separation of aortas.  Tissue was digested in RPMI containing 450 U/mL Collagenase Ⅰ, 125U/mL Collagenase Ⅺ and 60 U/mL Hyaluronidase at 37℃ for 40 minutes.  The suspension was filtered through a 40 µm strainer.  FACS sorted CD45^+^ cells. | 10x Genomics GemCode | Thirteen distinct clusters.  Myeloid cell subsets: resident-like macrophages (in healthy and atherosclerotic aortas); inflammatory macrophages, previously undescribed TREM2^hi^ macrophages, monocytes, and monocyte-derived dendritic cells (in atherosclerotic aortas). | Immunostaining detected enriched genes of inflammatory macrophages, res-like macrophages, and TREM2^hi^ macrophages in murine and human. atherosclerotic lesions. | (Cochain et al., 2018) |
| Mouse | CD11b^+^TdTomato^+^ cells of aortic arches from Cx3cr1^CreERT2-IRES-YFP/+^Rosa26^floxed-tdTomato/+^mice | Separation of aortic arches.  Tissue was digested in Dulbecco’s PBS (DPBS) containing 4 U/mL Liberase TH, 0.1 mg/mL DNase I and 60 U/ml Hyaluronidase at 37°C for 60 minutes.  FACS sorted CD11b^+^TdTomato^+^ cells. | 10x Genomics Chromium | A spectrum of macrophage activation states.  During atherosclerosis progression, CXC3R1^+^ monocytes differentiated into more distinct states than during regression.  A cluster of proliferating monocytes displaying a stem cell-like signature. | Immunofluorescence confirmed the existence of the stem cell–like proliferative CX3CR1^+^ cell cluster in atherosclerotic plaque. | (Lin et al., 2019) |
| Mouse | CD45^+^ cells of whole aortas from Ldlr^-/-^ mice  SSC^hi^BODIPY^hi^ cells of whole aortas from ApoE^-/-^ mice | Aortas were separated and cut into small pieces.  Tissue was digested in PBS with calcium and magnesium containing 675 U/mL Collagenase Ⅰ, 90 U/mL DNase Ⅰ, 187.5 U/mL Collagenase Ⅺ, and 90 U/mL Hyaluronidase at 37℃ for 70 minutes.  FACS sorted CD45^+^ cells or SSC^hi^BODIPY^hi^ cells. | 10x Genomics Chromium | Eleven leukocyte clusters, where macrophages covered the largest number of cells with high diversity.  Foamy macrophages expressed low levels of inflammatory genes.  CD45^−^ foam cells were derived from SMCs and ECs. | It was indicated by *in situ* hybridization that foamy CD68^+^ macrophages showed lower level of IL-1β mRNA compared with non-foamy macrophages.  Quantitative PCR (qPCR) analysis of foamy and intimal non-foamy macrophages indicated that non-foamy macrophages expressed *Il1β*, while foamy macrophages did not. | (Kim et al., 2018) |
| Mouse | CD45^+^ cells of aortic arches from Ntn1^fl/fl^Cx3cr1^creERT2+^ mice and Ntn1^fl/fl^Cx3cr1^WT^ mice | Aortic arches were separated and digested in Liberase (5 mg/mL), Hyaluronidase (99 ug/mL) and DNase I (58 ug/mL) for 15 min at 37°C using the GentleMacs dissociator.  FACS sorted CD45+ cells. | 10x Genomics Chromium | Thirteen distinct immune cell clusters, including one monocyte cluster and two macrophage clusters (Cx3cr1^−^ macrophages and TREM2^hi^ macrophages).  *Ntn1* silencing resulted in increasing expression of M2 marker genes and upregulation of gene pathways associated with macrophage phagocytosis and migration in monocytes and TREM2^hi^ macrophages. | Immunostaining and co-incubation of bone marrow-derived macrophages and apoptotic Jurkat cells confirmed that Ntn1silencing promotes a proresolving macrophage phenotype. | (Schlegel et al., 2021) |
| Mouse | CD45^+^CD11b^+^F4/80^+^ macrophages of aortic arches from mice in baseline  and regression groups | Separation of aortic arches.  Tissue was digested in DPBS containing 4 U/mL Liberase TH, 0.1 mg/mL DNase I and 60 U/ml Hyaluronidase at 37°C for 60 minutes.  FACS sorted CD45^+^CD11b^+^F4/80^+^ macrophages. | Fluidigm C1 | M2-like macrophages could be derived from newly recruited monocytes in regressing plaques. |  | (Rahman et al., 2017) |
| Mouse | CD45^+^ cells of aortic arches from hypercholesterolemic mice | Aortic arches were separated and digested in Liberase, Hyaluronidase and DNase I for15 min at 37°C using the GentleMacs dissociator.  FACS sorted CD45^+^ cells. | 10x Genomics Chromium | Pf4 and Ppbp expression increased in macrophages from plaques, suggesting increased macrophage-platelet aggregates in plaques.  In hypercholesterolemia, Socs3 expression increased, and the Socs1:Socs3 ratio decreased in atherosclerotic plaque macrophages. | *In vitro*, peritoneal macrophage exposing to different concentration of platelets showed a platelet concentration-dependent increase in Socs3 mRNA expression, and a significant decrease Socs1:Socs3 ratio. | (Barrett et al., 2019) |
| Mouse | CD45^+^ cells of aortic arch plaques from atherosclerotic Ldlr^−/−^ mice induced by adeno-associated virus -mediated delivery of PCSK9 or western diet | Aortic arches were separated and digested in Liberase, Hyaluronidase, DNase I and 1 M CaCl_2_ for15 min at 37°C using the GentleMacs dissociator.  FACS sorted CD45^+^ cells. | 10x Genomics Chromium | Compared with Tregs from progressing plaques showing natural markers, Tregs in regressing plaques lacked Nrp1 expression.  Tregs changed the landscape of plaque macrophages during regression. | Immunofluorescence staining of aortic root plaques from mice indicated that Treg cells promote M2-like macrophage enrichment, apoptotic cell clearance, fibrous cap formation, and production of inflammation-dampening cytokines. | (Sharma et al., 2020) |
| Mouse | ApoB^neg^ and ApoB^+^ cells of lymph nodes from ApoE^-/-^mice  CD45^+^ cells of aortas from ApoE^-/-^mice | FACS sorted ApoB^neg^ and ApoB^+^ cells from cell suspension of lymph nodes.  FACS sorted CD45^+^ cells from cell suspension of aortas. | 10x Genomics Chromium | Several clusters of ApoB^+^ T cells showed mixed T_H_ signatures.  T_H_17/T_H_1 cells increased in the atherosclerotic plaque. | It was confirmed by flow cytometry of blood from humans with or without coronary artery atherosclerosis (CAD) that ApoB-reactive T cells with a predominant T_H_17 and T_H_1 phenotype increase in humans with CAD. | (Wolf et al., 2020) |
| Mouse  Human | Cells of advanced brachiocephalic artery (BCA) lesions from Myh11-Cre^ERT2^Rosa-eYFP ApoE^-/-^ SMC lineage tracing mice, Cdh5-Cre^ERT2^Rosa-eYFP ApoE^-/-^ EC lineage tracing mice, SMCKlf4^-Δ/Δ^ ApoE^-/-^ mice, and SMCKlf4^-WT/WT^ ApoE^-/-^ mice  Cells of BCA plaques from Myh11-Dre^ERT2^ Lgals3-Cre Rosa-tdTomato-eGFP ApoE^-/-^ mice  Cells of human atherosclerotic plaques | Cells from mouse: Advanced BCA lesions were minced and digested with 1mL Liberase plus 1ug/mL Actinomycin D for 1 hour at 37C°, then resuspended in PBS plus 0.04% UltraPure nonacetylated BSA and filtered through 30um filters on ice.  Cells from human: Atherosclerotic plaques were minced and digested in RPMI 1640 containing 2.5 mg/mL Collagenase IV, 0.25 mg/mL DNAse I, 2.5 mg/mL Human Albumin Fraction V  and 1 mM Flavopiridol at 37 °C for 30 minutes.  Cell suspension was filtered through a 70 µm strainer. | 10x Genomics Chromium | Significant similarity in transcriptomic clusters between humans and mice.  Klf4 regulated SMC transition to multiple phenotypes, including Lgals3^+^ osteogenic cells. | Staining of sections from advanced mouse BCA lesions showed that the number of cells with osteogenic phenotype significantly decreased in SMC^Klf4-Δ/Δ^ mice versus SMC^Klf4-WT/WT^ mice. | (Alencar et al., 2020) |
| Mouse  Human | ZsGreen1^+^ and ZsGreen1^-^ cells of aortas from ROSA26^ZsGreen1/+^, Ldlr^-/-^, and Myh11-CreER^T2^ mice  ZsGreen1^+^ and ZsGreen1^-^ cells of aortas of ROSA26^ZsGreen1/+^, ApoE^-/-^, and Myh11-CreER^T2^ mice  Human atherosclerotic carotid arteries | Aortas were minced and digested in RPMI 1640 containing 4 U/mL Liberase TM, 60 U/mL hyaluronidase, and 120 U/mL DNase I at 37°C for 45 minutes.  Cell suspensions were filtered through 70 µm strainers.  FACS sorted ZsGreen1^+^ and ZsGreen1^-^ cells. | 10x Genomics Chromium | SMC-derived intermediate cells, termed "SEM" cells (stem cells, ECs, monocytes)  RA signaling was identified as a regulator of SMC-to-SEM cell transition. | RNAscope with a probe set targeting Vcam1 mRNA detected SEM cells in in atherosclerotic lesions.  ZsGreen1^+^ SEM cells from mouse atherosclerotic aortas could differentiated into macrophage-like and fibrochondrocyte-like cells and reverted towards SMC-like phenotype *in vitro*.  *In vitro* cell experiments, flow cytometry of atherosclerotic lesions and RNAscope of BCA sections confirmed that all-trans retinoic acid (ATRA), an activator of RA signaling, suppresses SMC to SEM cell transition. | (Pan et al., 2020) |
| Mouse  Human | TdTomato^+^ and tdTomato^-^ cells of aortic root and ascending aorta from SMC^lin^ and SMC^lin-KO^ mice (lin= lineage tracing, KO= specific conditional *Tcf21* knockout)  Cells of human atherosclerotic coronary arteries | Aortic roots and ascending aortas of mice were minced and digested in HBSS containing 2 U/mL Liberase TM, 2 U/mL elastase at 37 °C for1 hour. FACS sorted tdTomato^+^ and tdTomato^-^ cells.  Atherosclerotic coronary arteries of human were minced and digested in HBSS containing 10.4 U/mL Liberase TM, 8 U/mL elastase at 37 °C for 1 hour with periodic agitation. | 10x Genomics Chromium | The transcriptomic phenotype of modulated SMCs transformed into unique fibroblast-like cells, termed 'fibromyocytes'.  TCF21 expression was strongly associated with SMC phenotypic modulation. | RNAscope of atherosclerotic mouse aortic root and human lesions revealed the distribution of fibromyocytes. | (Wirka et al., 2019) |
| Mouse | TdTomato ^+^ cells of Aortic roots from SMC^Lin^ and SMC^Lin-KO^ mice on a high fat diet (lin= lineage tracing, KO= AHR knockout) | Aortic roots were minced and digested in RPMI 1640 containing 4U/mL Liberase TM, 0.1mg/mL DNaseI, and 60U/mL Hyaluronidase at 37°C for 1.5 hours.  Cell suspension was filtered through a 70 µm strainer.  FACS sorted TdTomato ^+^ cells. | 10x Genomics Chromium | The average expression of *Cyp1b1* was higher in fibromyocytes compared to SMC clusters, suggesting the higher activity of *Ahr* pathway in fibromyocytes.  Chondromyocytes, the modulated SMCs expressing chondrocyte markers (Col2a1 and Alpl), increased in SMC-specific *AHR*-knockout mice. | RNAscope in-situ hybridization confirmed that the *Ahr* pathway activity in the media and the lesion cap is present in fibromyocytes.  RNAscope, Alcian blue stain and alkaline phosphatase (AP) activity indicated that chondromyocytes locates in the intima of the lesions, and the number of chondromyocytes is greater in SMC^Lin-KO^ mice.  It was confirmed by primary human coronary artery smooth muscle cells with or without *Ahr* knockdown *in vitro* that Ahr has an anti-migratory, anti-proliferative, anti-calcifying, and pro-apoptotic effect. | (Kim et al., 2020) |
| Mouse | VSMC of medial layer of aortas from WT C57BL/6 mice and Myh11-CreERt2/Confetti mice;  Cells of medial layers from three tamoxifen-labelled Myh11-CreERt2/Confetti mice;  Cells of whole aortas and plaques from Myh11-CreERt2/Confetti/ApoE^-/-^ mice | Endothelial cells were removed and aortas were incubated for 10 minutes in DMEM containing 1 mg/mL Collagenase IV, and 1 U/mL porcine pancreatic elastase to separate adventitia and medial cell layers.  To obtain cell suspension, tissue was further digested for 1–2 hours.  Cell suspension was filtered through a 40 µm cell strainer. | Fluidigm C1  Smart-seq2  10x Genomics Chromium | A rare subset of VSMCs was detected, which expressed the multipotent progenitor marker Sca1.  Sca1 upregulation is a marker of VSMCs undergoing phenotypic transformation. | Flow cytometry and Sca1-GFP animals confirmed the existence of Sca1^+^ VSMCs. | (Dobnikar et al., 2018) |
| Human | Cells of atherosclerotic plaque of human carotid artery | Gene Expression Omnibus (GSE159677) | 10x Genomics Chromium | The expression of lysosome- and inflammation-related genes increased in VSMCs derived macrophage-like cells.  There was negative correlation between NOTCH genes and macrophage genes. | Immunostaining of human atherosclerotic plaque showed that media VSMCs proximal to plaque necrosis core expressing CD68.  QRT-PCR of primary rat VSMCs revealed that the expression of macrophage marker gene *Cd68* and *Lgals3* increased, while the expression of VSMC marker gene *Acat2* and *Tagln* decreased after cholesterol loading.  Immunostaining of VSMCs confirmed that NOTCH inhibition promoted the generation of Cd68^+^ cells. | (Zhang et al., 2022b) |
| Mouse | CD45^-^ cells of aortic arch and root and descending thoracic aorta from ApoE^-/-^ mice | Aortic arch and root and descending thoracic aorta were separated without adventitia.  Tissue was digested at 37 °C in DMEM containing Collagenase P, dispase and DNaseI.  Cell suspension was filtered through a 70 µm cell strainer.  FACS sorted CD45^-^ cells. | Fluidigm C1 | Five VSMCs phenotypes: macrophagic calcific, mesenchymal chondrogenic, inflammatory and fibrous, inflammatory, and preserved contractile phenotypes.  GDF10 promoted VCMS transition to an osteogenic phenotype. | Immunofluorescence staining revealed that the number of osteogenic-like VSMCs co-expressing GDF-10 and Alkaline Phosphatase, as well as GDF10 and RUNX2 (a marker of VSMC osteogenic switch), were significantly increased in the aortic root plaques of Apoe^−/−^Myh11-CreERT2, ROSA26STOP-floxeYFP^+/+^ mice with a high cholesterol diet versus Apoe^−/−^Myh11-CreERT2, ROSA26STOP-floxeYFP^+/+^ mice with a normal cholesterol diet. | (Brandt et al., 2022) |
| Human | Cells of Plaque samples from human carotid artery | Plaque samples minus the necrotic core were minced and digested.  The cell suspension was filtered through a 70 µm strainer and washed with RPMI 1640. Cells were kept in t RPMI 1640 with 1% fetal calf serum | SORT-seq  CEL-seq2 | The key drivers of gene regulatory networks active in female coronary artery disease were identified primarily in SMCs.  Female key drivers expressed by phenotypically modulated SMCs and influenced by Klf4. |  | (Hartman et al., 2021) |
| Mouse | EYFP^+^ cells of BCA plaques from SMC *Has3* WT and SMC *Has3*-knockout mice | BCA plaques were separated and transferred into FACS buffer (1% BSA in PBS) and 1 µg/mL Actinomycin D, and digested with 1 mL Liberase for 1 hour at 37 °C.  Cell suspensions were filtered through 30 µm filters on ice.  FACS sorted eYFP^+^ cells. | 10x Genomics Chromium | SMC *Has3* deletion promoted modulated SMC phenotypes (Lgals3^+^) and enhanced the acute-phase response. | It was confirmed that  Has3 targeted siRNA treatment and CD44 (a hyaluronan-binding receptor) blocking decreased the expression of *Acta2*, and increased the expression of *Col1a1*, *Col15a1*, *Col3a1* and *Fn1* in murine SMCs stimulated with PDGF-BB and TGF-β1. | (Hartmann et al., 2021) |
| Mouse | Cells of BCA region from Pdgfrb  ^SMC-Δ/Δ^ and Pdgfrb^SMC-WT/WT^ mice | Samples were minced and digested with Liberase and 1μg/mL Actinomycin-D for 1 hour at 37°C. | 10x Genomics Chromium | SMC-MF transitions were induced by PDGF and TFGβ and depended on aerobic glycolysis. | It was confirmed by *in vitro* experiments of murine SMCs that aerobic glycolysis is required for SMC transition induced by PDGF and TGFβ1. | (Newman et al., 2021) |
| Mouse  Human | ECs of hearts and aortas from Ldlr-/- mice | Hearts and whole aortas were dissected into small pieces and digested in DMEM containing 2mg/mL Collagenase I and 60 U/mL DNase I at 37°C for 30–45 minutes with shaking.  Cell suspension was filtered through a 40 µm strainer.  ECs were enriched by CD31 MicroBeads. | 10x Genomics Chromium | Eight EC clusters, including three clusters expressing mesenchymal markers indicative of EndMT.  EndMT-derived fibroblast-like cells are significant in atherosclerosis. |  | (Zhao et al., 2021) |
| Mouse | Cells of left and right carotid arteries from C57BL/6 mice with PCL | Dissociation buffer (600 U /mL Type II Collagenase and 60 U/mL DNase I in 0.5% FBS of PBS) was injected into carotid lumens.  The ends of left and right carotid arteries were clamped and dissected out, and incubated in HBSS at 37 °C. After 45 minutes, the lumens were flushed with the dissociation buffer into a 1.5 mL Eppendorf tube. | 10x Genomics Chromium | Eight EC clusters with high heterogeneity and plasticity  D-flow induces EC transformation from an atheroprotective to a proatherogenic phenotype. | It was confirmed by cultured human aortic ECs *in vitro* and immunostaining of mouse carotid arteries that d-flow induces expression of immune cell markers in ECs. | (Andueza et al., 2020) |
| Mouse | Cells of left and right carotid arteries from C57BL/6 mice with PCL | Dissociation buffer (600 U /mL Type II Collagenase and 60 U/mL DNase I in 0.5% FBS of PBS) was injected into carotid lumens.  The ends of left and right carotid arteries were clamped and dissected out, and incubated in HBSS at 37 °C. After 45 minutes, the lumens were flushed with the dissociation buffer into a 1.5 mL Eppendorf tube. | 10x Genomics Chromium | Klk10 expression was regulated by flow in ECs. | Immunostaining, qPCR, western blots, and ELISA *in vivo* and *in vitro* confirmed that KLK10 protein and mRNA expression were significantly higher in ECs in the s-flow right carotid artery compared to the d-flow left carotid artery. | (Williams et al., 2022) |
| Mouse | Cells of left carotid arteries from C57BL/6 mice with or without PCL | Left carotid arteries were chopped and incubated in a solution containing 1 mg/mL Collagenase Type II, 0.02 mg/mL Deoxyribonuclease I for 1 h at 37 °C.  Cell suspension was filtered through a 40 µm strainer. | 10x Genomics Chromium | Fifteen distinct clusters, including 10 d-flow-relevant subsets.  Dkk2^hi^ EC subpopulation probably transformed from Klk8^hi^ ECs because of d-flow.  Spp1hi VSMC subpopulation induced by d-flow showed osteoblast signatures.  Birc5^hi^ macrophages was considered a potential factor of macrophage aggregation in atherosclerosis. | Immunofluorescence staining detected the existence of Birc5^hi^ macrophages or Trem2^hi^ macrophages after PCL.  Immunostaining revealed that Cd36^hi^ ECs and Dkk2^hi^ ECs exist in the region where the d-flow occurs in WT or atherosclerotic mice. | (Li et al., 2021) |
| Mouse | Cells of aortas from ApoE^-/-^ mice | Aortas were separated and digested in in HBSS containing 2U/mL Liberase TM and 2U/mL Elastase at 37°C for 1 hour.  Cell suspension was filtered through a 70 µm strainer. | 10x Genomics Chromium | Pro-efferocytic SWNT decreased the expression of inflammatory genes in lesional macrophages. |  | (Flores et al., 2020) |
| Mouse | Cells of aortas from anti-miR-33^pHLIP^–treated and Scr^pHLIP^-treated Ldlr^−/−^ mice | Aorta was digested with 1 mg/mL Collagenase A for 7 minutes at 37 °C to remove the adventitia.  Tissue was minced and digested in 1.5 mg/mL Collagenase A and 0.5 mg/mL Elastase for 40 minutes at 37 °C with shaking.  Cell suspension was filtered through a 70 µm strainer. | 10× Genomics GemCode | Five aortic monocyte and macrophage populations: F10^+^ monocytes and Trem2^high^, inflammatory, stem-like, and ECM^high^ macrophages.  Anti-miR-33^pHLIP^ increased the percentage of ECM^high^ and stem-like macrophages and decreased the percentage of inflammatory macrophages.  Anti-miR-33^pHLIP^ upregulated fibrosis, M2 polarization, and antigen presentation pathways. The expression of fibrotic genes (*Col2a1, Col3a1, Col1a2,* and *Fn1*) and *Timp3* increased in macrophages from aortas from anti-miR-33^pHLIP^–treated mice, while *Mmp12* expression decreased. |  | (Zhang et al., 2022a) |
| Mouse  Human | CD45^+^ cells of plaques from ApoE^-/-^ mice  Cells of human plaques | Plaques of mouse were separated and cut into small pieces, then digested in DPBS containing 400 U/mL Collagenase Type I, 10 U/mL Collagenase Type XI, 60 U/mL Hyaluronidase, 60 U/mL DNase I and 20 mM HEPES for 40 minutes at 37°C with shaking.  FACS sorted CD45^+^ cells.  Raw data of human plaques single cell sequencing was downloaded from a publicly available databank (<https://figshare.com/s/c00d88b1b25ef0c5c788>). | 10x Genomics | The *Gal3* expression pattern in human plaques was similar to that in mouse plaques. |  | (Varasteh et al., 2021) |

**References**

Alencar, G.F., Owsiany, K.M., Karnewar, S., Sukhavasi, K., Mocci, G., Nguyen, A.T., et al. (2020). Stem Cell Pluripotency Genes Klf4 and Oct4 Regulate Complex SMC Phenotypic Changes Critical in Late-Stage Atherosclerotic Lesion Pathogenesis. *Circulation* 142(21)**,** 2045-2059. doi: 10.1161/circulationaha.120.046672.

Andueza, A., Kumar, S., Kim, J., Kang, D.W., Mumme, H.L., Perez, J.I., et al. (2020). Endothelial Reprogramming by Disturbed Flow Revealed by Single-Cell RNA and Chromatin Accessibility Study. *Cell Rep* 33(11)**,** 108491. doi: 10.1016/j.celrep.2020.108491.

Barrett, T.J., Schlegel, M., Zhou, F., Gorenchtein, M., Bolstorff, J., Moore, K.J., et al. (2019). Platelet regulation of myeloid suppressor of cytokine signaling 3 accelerates atherosclerosis. *Sci Transl Med* 11(517). doi: 10.1126/scitranslmed.aax0481.

Brandt, K.J., Burger, F., Baptista, D., Roth, A., Fernandes da Silva, R., Montecucco, F., et al. (2022). Single-Cell Analysis Uncovers Osteoblast Factor Growth Differentiation Factor 10 as Mediator of Vascular Smooth Muscle Cell Phenotypic Modulation Associated with Plaque Rupture in Human Carotid Artery Disease. *Int J Mol Sci* 23(3). doi: 10.3390/ijms23031796.

Cochain, C., Vafadarnejad, E., Arampatzi, P., Pelisek, J., Winkels, H., Ley, K., et al. (2018). Single-Cell RNA-Seq Reveals the Transcriptional Landscape and Heterogeneity of Aortic Macrophages in Murine Atherosclerosis. *Circ Res* 122(12)**,** 1661-1674. doi: 10.1161/circresaha.117.312509.

Depuydt, M.A.C., Prange, K.H.M., Slenders, L., Örd, T., Elbersen, D., Boltjes, A., et al. (2020). Microanatomy of the Human Atherosclerotic Plaque by Single-Cell Transcriptomics. *Circ Res* 127(11)**,** 1437-1455. doi: 10.1161/circresaha.120.316770.

Dobnikar, L., Taylor, A.L., Chappell, J., Oldach, P., Harman, J.L., Oerton, E., et al. (2018). Disease-relevant transcriptional signatures identified in individual smooth muscle cells from healthy mouse vessels. *Nat Commun* 9(1)**,** 4567. doi: 10.1038/s41467-018-06891-x.

Flores, A.M., Hosseini-Nassab, N., Jarr, K.U., Ye, J., Zhu, X., Wirka, R., et al. (2020). Pro-efferocytic nanoparticles are specifically taken up by lesional macrophages and prevent atherosclerosis. *Nat Nanotechnol* 15(2)**,** 154-161. doi: 10.1038/s41565-019-0619-3.

Gu, W., Ni, Z., Tan, Y.Q., Deng, J., Zhang, S.J., Lv, Z.C., et al. (2019). Adventitial Cell Atlas of wt (Wild Type) and ApoE (Apolipoprotein E)-Deficient Mice Defined by Single-Cell RNA Sequencing. *Arterioscler Thromb Vasc Biol* 39(6)**,** 1055-1071. doi: 10.1161/atvbaha.119.312399.

Hartman, R.J.G., Owsiany, K., Ma, L., Koplev, S., Hao, K., Slenders, L., et al. (2021). Sex-Stratified Gene Regulatory Networks Reveal Female Key Driver Genes of Atherosclerosis Involved in Smooth Muscle Cell Phenotype Switching. *Circulation* 143(7)**,** 713-726. doi: 10.1161/circulationaha.120.051231.

Hartmann, F., Gorski, D.J., Newman, A.A.C., Homann, S., Petz, A., Owsiany, K.M., et al. (2021). SMC-Derived Hyaluronan Modulates Vascular SMC Phenotype in Murine Atherosclerosis. *Circ Res* 129(11)**,** 992-1005. doi: 10.1161/circresaha.120.318479.

Kim, J.B., Zhao, Q., Nguyen, T., Pjanic, M., Cheng, P., Wirka, R., et al. (2020). Environment-Sensing Aryl Hydrocarbon Receptor Inhibits the Chondrogenic Fate of Modulated Smooth Muscle Cells in Atherosclerotic Lesions. *Circulation* 142(6)**,** 575-590. doi: 10.1161/circulationaha.120.045981.

Kim, K., Shim, D., Lee, J.S., Zaitsev, K., Williams, J.W., Kim, K.W., et al. (2018). Transcriptome Analysis Reveals Nonfoamy Rather Than Foamy Plaque Macrophages Are Proinflammatory in Atherosclerotic Murine Models. *Circ Res* 123(10)**,** 1127-1142. doi: 10.1161/circresaha.118.312804.

Li, F., Yan, K., Wu, L., Zheng, Z., Du, Y., Liu, Z., et al. (2021). Single-cell RNA-seq reveals cellular heterogeneity of mouse carotid artery under disturbed flow. *Cell Death Discov* 7(1)**,** 180. doi: 10.1038/s41420-021-00567-0.

Lin, J.D., Nishi, H., Poles, J., Niu, X., McCauley, C., Rahman, K., et al. (2019). Single-cell analysis of fate-mapped macrophages reveals heterogeneity, including stem-like properties, during atherosclerosis progression and regression. *JCI Insight* 4(4). doi: 10.1172/jci.insight.124574.

Newman, A.A.C., Serbulea, V., Baylis, R.A., Shankman, L.S., Bradley, X., Alencar, G.F., et al. (2021). Multiple cell types contribute to the atherosclerotic lesion fibrous cap by PDGFRβ and bioenergetic mechanisms. *Nat Metab* 3(2)**,** 166-181. doi: 10.1038/s42255-020-00338-8.

Pan, H., Xue, C., Auerbach, B.J., Fan, J., Bashore, A.C., Cui, J., et al. (2020). Single-Cell Genomics Reveals a Novel Cell State During Smooth Muscle Cell Phenotypic Switching and Potential Therapeutic Targets for Atherosclerosis in Mouse and Human. *Circulation* 142(21)**,** 2060-2075. doi: 10.1161/circulationaha.120.048378.

Rahman, K., Vengrenyuk, Y., Ramsey, S.A., Vila, N.R., Girgis, N.M., Liu, J., et al. (2017). Inflammatory Ly6Chi monocytes and their conversion to M2 macrophages drive atherosclerosis regression. *J Clin Invest* 127(8)**,** 2904-2915. doi: 10.1172/jci75005.

Schlegel, M., Sharma, M., Brown, E.J., Newman, A.A.C., Cyr, Y., Afonso, M.S., et al. (2021). Silencing Myeloid Netrin-1 Induces Inflammation Resolution and Plaque Regression. *Circ Res* 129(5)**,** 530-546. doi: 10.1161/circresaha.121.319313.

Sharma, M., Schlegel, M.P., Afonso, M.S., Brown, E.J., Rahman, K., Weinstock, A., et al. (2020). Regulatory T Cells License Macrophage Pro-Resolving Functions During Atherosclerosis Regression. *Circ Res* 127(3)**,** 335-353. doi: 10.1161/circresaha.119.316461.

Varasteh, Z., De Rose, F., Mohanta, S., Li, Y., Zhang, X., Miritsch, B., et al. (2021). Imaging atherosclerotic plaques by targeting Galectin-3 and activated macrophages using ((89)Zr)-DFO- Galectin3-F(ab')(2) mAb. *Theranostics* 11(4)**,** 1864-1876. doi: 10.7150/thno.50247.

Williams, D., Mahmoud, M., Liu, R., Andueza, A., Kumar, S., Kang, D.W., et al. (2022). Stable flow-induced expression of KLK10 inhibits endothelial inflammation and atherosclerosis. *Elife* 11. doi: 10.7554/eLife.72579.

Winkels, H., Ehinger, E., Vassallo, M., Buscher, K., Dinh, H.Q., Kobiyama, K., et al. (2018). Atlas of the Immune Cell Repertoire in Mouse Atherosclerosis Defined by Single-Cell RNA-Sequencing and Mass Cytometry. *Circ Res* 122(12)**,** 1675-1688. doi: 10.1161/circresaha.117.312513.

Wirka, R.C., Wagh, D., Paik, D.T., Pjanic, M., Nguyen, T., Miller, C.L., et al. (2019). Atheroprotective roles of smooth muscle cell phenotypic modulation and the TCF21 disease gene as revealed by single-cell analysis. *Nat Med* 25(8)**,** 1280-1289. doi: 10.1038/s41591-019-0512-5.

Wolf, D., Gerhardt, T., Winkels, H., Michel, N.A., Pramod, A.B., Ghosheh, Y., et al. (2020). Pathogenic Autoimmunity in Atherosclerosis Evolves From Initially Protective Apolipoprotein B(100)-Reactive CD4(+) T-Regulatory Cells. *Circulation* 142(13)**,** 1279-1293. doi: 10.1161/circulationaha.119.042863.

Zhang, X., Rotllan, N., Canfrán-Duque, A., Sun, J., Toczek, J., Moshnikova, A., et al. (2022a). Targeted Suppression of miRNA-33 Using pHLIP Improves Atherosclerosis Regression. *Circ Res***,** 101161circresaha121320296. doi: 10.1161/circresaha.121.320296.

Zhang, Z., Huang, J., Wang, Y., and Shen, W. (2022b). Transcriptome analysis revealed a two-step transformation of vascular smooth muscle cells to macrophage-like cells. *Atherosclerosis* 346**,** 26-35. doi: 10.1016/j.atherosclerosis.2022.02.021.

Zhao, G., Lu, H., Liu, Y., Zhao, Y., Zhu, T., Garcia-Barrio, M.T., et al. (2021). Single-Cell Transcriptomics Reveals Endothelial Plasticity During Diabetic Atherogenesis. *Front Cell Dev Biol* 9**,** 689469. doi: 10.3389/fcell.2021.689469.
